# Post-acute sequelae of COVID-19 in a non-hospitalized cohort: Results from the Arizona CoVHORT

**DOI:** 10.1371/journal.pone.0254347

**Published:** 2021-08-04

**Authors:** Melanie L. Bell, Collin J. Catalfamo, Leslie V. Farland, Kacey C. Ernst, Elizabeth T. Jacobs, Yann C. Klimentidis, Megan Jehn, Kristen Pogreba-Brown

**Affiliations:** 1 Department of Epidemiology and Biostatistics, Mel and Enid Zuckerman College of Public Health, The University of Arizona, Tucson, AZ, United States of America; 2 School of Human Evolution and Social Change, Arizona State University, Tempe, AZ, United States of America; Mayo Clinic Minnesota, UNITED STATES

## Abstract

Clinical presentation, outcomes, and duration of COVID-19 has ranged dramatically. While some individuals recover quickly, others suffer from persistent symptoms, collectively known as long COVID, or post*-*acute sequelae of SARS-CoV-2 (PASC). Most PASC research has focused on hospitalized COVID-19 patients with moderate to severe disease. We used data from a diverse population-based cohort of Arizonans to estimate prevalence of PASC, defined as experiencing at least one symptom 30 days or longer, and prevalence of individual symptoms. There were 303 non-hospitalized individuals with a positive lab-confirmed COVID-19 test who were followed for a median of 61 days (range 30–250). COVID-19 positive participants were mostly female (70%), non-Hispanic white (68%), and on average 44 years old. Prevalence of PASC at 30 days post-infection was 68.7% (95% confidence interval: 63.4, 73.9). The most common symptoms were fatigue (37.5%), shortness-of-breath (37.5%), brain fog (30.8%), and stress/anxiety (30.8%). The median number of symptoms was 3 (range 1–20). Amongst 157 participants with longer follow-up (≥60 days), PASC prevalence was 77.1%.

## Introduction

Clinical presentation and outcomes of infection with severe acute respiratory syndrome coronavirus 2 (SARS-CoV-2) range from asymptomatic infection to death [1]. Duration of illness has also varied; some individuals recover quickly, but others experience persistent, often debilitating, symptoms. The experience of symptoms lasting 30 days and longer has been dubbed long COVID, or post*-*acute sequelae of COVID*-*19 (PASC). Currently, the scientific community’s understanding of PASC is still evolving, and understanding of the diagnoses, phenotypes and epidemiology is nascent [2]. Most research to date has focused on PASC amongst hospitalized patients [3]. However, most individuals with COVID-19 are not hospitalized, and PASC among these non-hospitalized individuals is not as well characterized. We aimed to describe the prevalence of post-COVID-19 symptoms amongst individuals who experienced mild-to-moderate COVID-19 using data from a diverse, population-based cohort.

## Methods

Written informed consent was obtained from all participants. Ethics approval was obtained from the University of Arizona Institutional Review Board (Protocol #2003521636A002). In May 2020, we began recruitment for CoVHORT, a prospective cohort study aimed to investigate the impacts of the SARS-Cov-2 pandemic among Arizona residents [4]. Briefly, targeted recruitment into CoVHORT occurs through multiple routes including enrollment of confirmed COVID-19 participants occurred via case investigations for Arizona health departments conducted by The University of Arizona’s Student Aid for Field Epidemiology Response program [5]. To recruit a representative sample of the community, several different recruitment strategies were utilized including invitations to participants of partnered COVID-19 studies, phased mailing campaigns, partnerships with community testing labs, and distribution of informational materials. All CoVHORT participants are emailed links to online surveys at 3 months, 6 months, 9 months, and 12 months post-baseline. At each of these surveys, we ask participants to provide updates to their SARS-CoV-2 infection and COVID-19 disease status including results of any diagnostic (PCR, antigen or antibody) tests completed since their last survey and information regarding the acute phase of infection if they have tested positive [4]. Participants who have tested positive are emailed a link to complete an additional online survey to assess the post-acute phase of their COVID-19 disease at 6 weeks post-report date of incident COVID-19, which asks whether they were experiencing any of 25 new or recurring symptoms (an open field was included for symptoms not listed). Because there is variability in how soon participants enroll in the CoVHORT study or complete their next follow-up survey after testing positive for a SARS-CoV-2 infection, participants’ follow-up time at the post-acute survey delivered at 6 weeks post-report of incident COVID is often longer than 42 days.

The current study included participants who had a positive polymerase chain reaction (PCR) or antigen test for SARS-COV-2 and provided symptom data at the 6 week assessment survey (see S1 File for the survey). We excluded 33 participants who reported that they were hospitalized in order to focus on less severe cases. We defined PASC as the presence of at least one symptom 30 days or longer following a positive test. Demographics, existing chronic conditions, COVID-19 acute-disease severity, and symptom data were self-reported.

### Statistical methods

Among COVID-19 positive participants, descriptive statistics were calculated by stratifying on PASC status, and compared using chi-square, t-tests, or non-parametric analogues. We calculated the prevalence and 95% confidence interval (CI) of PASC, number of symptoms, and self-reported COVID-19 related symptoms at ≥30, 30–59, and ≥ 60 days follow-up. We used Wald CIs for binary variables and distribution-free CIs for medians. We performed two sensitivity analyses. First, to account for potential dropout bias, where participants lost to follow-up may be less likely to experience PASC, we used multiple imputation (MI) with a delta adjustment, decreasing the likelihood of dropouts experiencing PASC by delta = 25%, 50%, and 75% [6]. MI models included factors associated with dropout. Second, because stress/anxiety is a symptom that many persons may be experiencing due to the pandemic, and not due to COVID-19 *per se*, we quantified the number of individuals who experienced stress/anxiety as their only symptom. All analyses were performed in SAS version 9.4 (Cary, NC).

## Results

Of the 3,468 participants in the CoVHORT as of 2/24/2021, 747 had a positive PCR or antigen test and were not hospitalized for their illness. Excluding participants who had incomplete COVID-19 testing information, 543 received a follow-up survey. Of these 543, 303 (55.8%) completed the follow-up surveys. Participants had a mean age of 44 years (range 12–82 years), were mostly female (70%), non-Hispanic white (68%), with college or greater education (38%), and with at least one pre-existing chronic condition (67%). The most commonly reported pre-existing conditions were seasonal allergies (42%), asthma (16%) and hypertension (15%). The overall mean self-reported severity rating of their COVID-19 acute illness was 4.6 out of 10. Individuals with PASC were more likely to have less education, at least one pre-existing chronic condition, seasonal allergies, and greater self-reported severity as compared to participants not experiencing PASC (Table 1). Females were more likely to experience PASC than males (73% versus 63%); however, this did not reach the threshold of statistical significance (p = 0.07).

**Table 1:** Characteristics of 303 Arizona CoVHORT participants with lab confirmed positive COVID-19 test, with 30 or more days of follow-up, from 28 May 2020 to 24 February 2021. Values are n (%) unless indicated otherwise.

Characteristic	Total (n = 303)	PASC (n = 208)	No PASC (n = 95)	p-value^a^
Follow-up (d), median (IQR)	61 (50, 85)	62 (53, 88)	57 (48, 80)	0.005
Age (y), mean (SD)	43.6 (16.6)	43.8 (15.8)	43.1 (18.2)	0.73
Female gender, n(%)	212 (70.0)	152 (73.1)	60 (63.2)	0.07
Race/ethnicity				0.70
Hispanic	69 (22.8)	50 (24.0)	19 (20.0)	
Non-Hispanic White	205 (67.7)	139 (66.8)	69 (72.6)	
Other race	19 (6.3)	14 (6.7)	5 (5.3)	
Education				0.0009
Some college or less	44 (14.5)	39 (18.8)	5 (5.3)	
College or more	115 (38.0)	72 (34.6)	43 (45.3)	
Missing	144 (47.5)	97 (46.6)	47 (49.5)	
BMI (kg/m^2^), mean (SD)	28.0 (7.0)	28.5 (7.3)	26.9 (6.2)	0.08
Currently smokes or vapes	40 (12.7)	27 (12.2)	13 (13.7)	0.72
Any pre-existing chronic condition ^b^	203 (67.0)	148 (71.2)	55 (57.9)	0.02
Seasonal allergies	127 (41.9)	97 (46.6)	30 (31.6)	0.01
Asthma	48 (15.8)	31 (14.9)	17 (17.9)	0.51
Hypertension	45 (14.9)	34 (16.3)	10 (10.5)	0.28
COVID-19 severity^c^, mean (SD)	4.6 (2.4)	5.1 (2.4)	3.4 (1.9)	<0.0001

Percentages may not add to 100% due to rounding and missing data. BMI = body mass index. PASC = post-acute sequalae of COVID-19, defined as any symptom reported at ≥30 days follow-up.

^a^ For the test of any reported symptom vs no reported symptom amongst non-missing values.

^b^ Self-reported pre-existing chronic conditions include: Thyroid disorder, asthma, chronic obstructive pulmonary disease, valley fever, emphysema, seasonal allergies, diabetes, pre-diabetes, cancer, heart disease, high blood pressure, liver disease, kidney disease, gastrointestinal conditions, depression/anxiety, autoimmune diseases.

^c^ Self-reported COVID-19 severity based on a scale from 1–10.

At ≥30 days follow-up, 208 of 303 (68.7%, 95% CI: 63.4, 73.9) COVID-19 positive participants reported experiencing PASC. The median number of symptoms among people experiencing PASC was 3 (range 1–20), with a median follow-up of 63 days (range 30–250). The 10 most commonly reported symptoms among individuals with PASC at ≥30 days post-positive test were fatigue (37.5%); shortness of breath (37.5%); brain fog (30.8%); stress/anxiety (30.8%); altered smell or taste (26.4%); body aches or muscle pains (26.0%); insomnia (22.1%); headaches (20.7%); joint pain (20.2%); and congestion or runny nose (19.2%) (Table 2).

**Table 2:** Self-reported symptoms of COVID-19 positive participants experiencing PASC at 30 or more days of follow-up from 28 May 2020–24 February 2021 in Arizona.

	Follow-up ≥30 days		Follow-up 30–59 days		Follow-up ≥60 days	
	N = 208		N = 87		N = 121	
	Estimate	95% CI	Estimate	95% CI	Estimate	95% CI
Number of symptoms, median (IQR)	3 (1, 6)	2, 4	3 (1, 5)	2, 4	3 (2, 7)	2, 4
Follow-up (d), median (IQR)	63 (53, 87.5)	60.0, 68.6	51 (48, 55)	49.9, 53.0	83 (68, 121)	77.0, 91.0
**Symptom, N (%)**						
Fatigue	78 (37.5)	30.9, 44.1	21 (24.1)	15.1, 33.1	57 (47.1)	38.2, 56.0
Shortness of breath	78 (37.5)	30.9, 44.1	23 (26.4)	17.2, 35.7	55 (45.5)	36.6, 54.3
Confusion or brain fog	64 (30.8)	24.5, 37.0	24 (27.6)	18.2, 37.0	40 (33.1)	24.7, 41.4
Stress and/or anxiety	64 (30.8)	24.5, 37.0	23 (26.4)	17.2, 35.7	41 (33.9)	25.5, 42.3
Changes in smell or taste	55 (26.4)	20.4, 32.4	25 (28.7)	19.2, 38.2	30 (24.8)	17.1, 32.5
Body aches or muscle pains	54 (26.0)	20.0, 31.9	17 (19.5)	11.2, 27.9	37 (30.6)	22.4, 38.8
Insomnia	46 (22.1)	16.5, 27.8	19 (21.8)	13.2, 30.5	27 (22.3)	14.9, 29.7
Headaches	43 (20.7)	15.2, 26.2	17 (19.5)	11.2, 27.9	26 (21.5)	14.2, 28.8
Joint pain	42 (20.2)	14.7, 25.6	15 (17.2)	9.3, 25.2	27 (22.3)	14.9, 29.7
Congestion or runny nose	40 (19.2)	13.9, 24.6	21 (24.1)	15.1, 33.1	19 (15.7)	9.2, 22.2
Gastrointestinal ^a^	38 (18.3)	13.0, 23.5	15 (17.2)	9.3, 25.2	23 (19.0)	12.0, 26.0
Faster than normal heart rate (Tachycardia)	36 (17.3)	12.2, 22.4	13 (14.9)	7.5, 22.4	23 (19.0)	12.0, 26.0
Cough	32 (15.4)	10.5, 20.3	11 (12.6)	5.7, 19.6	21 (17.4)	10.6, 24.1
Rash/skin conditions ^b^	32 (15.4)	10.5, 20.3	12 (13.8)	6.5, 21.0	20 (16.5)	9.9, 23.1
Dizziness/lightheadedness	26 (12.5)	8.0, 17.0	10 (11.5)	4.8, 18.2	16 (13.2)	7.2, 19.3
Chest pain or tightness	31 (14.9)	10.1, 19.7	13 (14.9)	7.5, 22.4	18 (14.9)	8.5, 21.2
High blood pressure	23 (11.1)	6.8, 15.3	6 (6.9)	1.6, 12.2	17 (14.1)	7.9, 20.2
Ringing in ears (Tinnitus)	19 (9.1)	5.2, 13.0	6 (6.9)	1.6, 12.2	13 (10.7)	5.2, 16.3
Menstrual issues ^c^	13 (8.6)	4.6, 14.2	8 (12.5)	5.6, 23.2	5 (5.7)	1.9, 12.8
Chills or sweats	17 (8.2)	4.5, 11.9	5 (5.8)	0.9, 10.6	12 (9.9)	4.6, 15.2
Loss of appetite	16 (7.7)	4.1, 11.3	8 (9.2)	3.1, 15.3	8 (6.6)	2.2, 11.0
Sore throat	15 (7.2)	3.7, 10.7	8 (9.2)	3.1, 15.3	7 (5.8)	1.6, 9.9
Changes in vision	13 (6.3)	3.0, 9.5	4 (4.6)	0.2, 9.0	9 (7.4)	2.8, 12.1
Pink eye	9 (4.3)	1.6, 7.1	1 (1.2)	0.0, 3.4	8 (6.6)	2.2, 11.0
Fever	8 (3.9)	1.2, 6.5	1 (1.2)	0.0, 3.4	7 (5.8)	1.6, 9.9
Hair loss	5 (2.4)	0.3, 4.5	0 (0.0)	0.0, 0.0	5 (4.1)	0.6, 7.7
Other ^d^	2 (1.0)	0.0, 2.3	0 (0.0)	0.0, 0.0	2 (1.7)	0.0, 3.9
Erectile dysfunction	1 (0.5)	0.0, 1.4	0 (0.0)	0.0, 0.0	1 (0.8)	0.0, 2.4

Abbreviations: IQR = interquartile range, CI = confidence interval, PASC = post-acute sequalae of COVID-19, defined as any symptom reported at ≥30 days follow-up.

^a^ Includes diarrhea, constipation, abdominal pain, nausea, vomiting.

^b^ COVID toes, hive-like rashes, skin discoloration, bruising.

^c^ Denominator includes women only (n = 152).

^d^ Other includes write-in symptoms not classified.

Among participants followed for 30–59 days post-diagnosis, 87 (59.6%, 95% CI: 51.6, 67.5) reported PASC and 121 (77.1% 95% CI: 70.5, 83.6) at ≥60 days follow-up. Frequencies of symptoms were higher at longer follow-up; though, the most prevalent symptoms were similar: fatigue, shortness-of-breath, and brain fog. We found that only 6 participants reported stress/anxiety as their sole symptom, demonstrating that our PASC prevalence is not driven by this non-specific symptom.

Compared to eligible participants with follow-up data, those without were younger (39 versus 44 years), more likely to be male (37 versus 30%), of Hispanic ethnicity (32 versus 23%), more likely to smoke or vape (25 versus 13%) and have lower education (60 versus 72% who had finished college). Disease severity rating was similar (4.9 versus 4.7 out of 10) as were rates of pre-existing conditions (64 versus 70%). In sensitivity analyses attempting to account for differences in follow-up using a not-at-random MI model, we estimated a PASC prevalence of 68.0% (delta = 0%), 58.8% (delta = 25%), 50.3% (delta = 50%) and 43.9% (delta = 75%), where delta is the decrease in likelihood of PASC for imputed values.

## Discussion

Among non-hospitalized lab confirmed COVID-19 positive participants, 68.7% experienced at least one symptom 30 days or longer past test-date. For individuals with ≥60 days follow-up, the prevalence of PASC was 73%. The most common symptoms were fatigue (37.5%), shortness of breath (37.5%), brain fog (30.8%), and stress/anxiety (30.8%). We found that less education, having at least one pre-existing condition (in particular, seasonal allergies), and greater self-reported COVID-19 severity at enrollment were associated with higher prevalence of PASC. While higher prevalence of PASC was seen among women and smokers/vapers, these were not statistically significant. Notably, we did not observe different prevalence estimates of PASC based on age or BMI.

Our estimated prevalence of PASC is only slightly less than prevalence estimates reported for hospitalized individuals: Huang et al. found a prevalence of 76% at 6 months [7]; Carfi et al. reported PASC prevalence of 87% at 2 months [3]. A systematic review of 7 studies of mixed follow-up and severity (hospitalized and non-hospitalized) by Lopez-Leon et al. found a PASC prevalence of 80% [8]. Studies that have included less severe cases have found lower estimates. Logue et al. reported prevalence of 33% among outpatients [9], Haverall et al. estimated a prevalence of 26% amongst mild cases [10] and Sudre et al., reported 13% in a mixed-severity group [11]. Logue et al.’s prevalence of 33% could possibly be due to their longer follow-up of 169 days, as compared to our median follow-up time of 59 days [9]. Their estimate of 33% is smaller than our most extreme sensitivity analysis (44%), where imputed values had a 75% reduced likelihood of experiencing PASC. Differences could also be due to the evolving definition of PASC [2]. A large study using Veteran Administration electronic health records reported increased risk of death, morbidity, heath resource utilization, and medication use among individuals who survived at least 30 days following COVID-19 diagnosis [12]. Their lower prevalence estimates are likely due to their use of electronic health records as compared to our self-report.

Our most common symptoms were similar to results from prior research; however, our prevalence of specific symptoms were lower than for studies with hospitalized participants. Commonly reported symptoms across multiple studies have been fatigue, headache, attention disorder (presumably similar to our variable, brain fog), hair loss, shortness of breath, sleeping problems, joint pain, dyspnea, chest pain and loss of sense of smell or taste [7–9, 13].

A strength of our study is that our sample is derived from non-hospitalized lab confirmed COVID-19 positive individuals recruited from public health surveillance efforts, a population in which PASC is not well-described. We also have a sizable proportion of Hispanics (23%) which is important, as minority and ethnic groups have been disproportionately affected by COVID-19 [2]. Our study also has limitations. Our response rate at follow-up was low (56%), and participants who completed follow-up questionnaires may differ from those who did not, possibly with a bias towards people who suffer from PASC. However, reported severity and having a pre-existing condition, both of which were associated with PASC, were similar between participants with and without follow-up. Furthermore, we conducted a statistically principled sensitivity analysis for loss to follow-up, and still estimated a high prevalence of PASC (47.7% (delta = 75%)). It is also possible that individuals suffering from severe PASC were too ill to complete follow-up surveys. Our characterization of the PASC phenotype is currently limited to 25 symptoms; other researchers have included more symptoms in their assessment [13]. All data were self-reported, and thus may be prone to recall bias and the subjective assessment of symptoms. Finally, we may have been underpowered to detect differences.

COVID-19 has infected more than 110,000,000 individuals worldwide as of 1 March 2021 [14]. If 63% (the lower limit of our 95% CI) of survivors experience persistent symptoms, over 63,000,000 individuals could be affected by the long-term consequences of COVID-19. Our estimate at ≥60 days follow-up showed that 50% of our participants were suffering from of 3 or more symptoms, with 25% experiencing 7 or more symptoms. These figures portend a public health challenge of massive scale. Further research is needed to characterize the clinical spectrum of PASC more completely in a variety of populations, including investigating correlates of PASC, treatment options, and time to resolution.

## Supporting information

S1 File (DOCX)
