# Supplementary material for: Post-acute sequelae of COVID-19 in a non-hospitalized cohort: Results from the Arizona CoVHORT
Source: PLoS One. 2021 Aug 4;16(8):e0254347. doi: 10.1371/journal.pone.0254347 (PMC8336814; doi:10.1371/journal.pone.0254347)
Supplement: S1 File — (DOCX) [file pone.0254347.s001.docx]

S1 File

# Demographics and baseline characteristics

Gender

- Male
- Female
- Non-binary
- Transgender female
- Transgender male
- Two spirit
- Prefer not to answer

What is your age?

- __________ yrs

What is your race?

- **American Indian or Alaska Native**. A person having origins in any of the original peoples of North and South America (including Central America), and who maintains tribal affiliation or community attachment
- **Asian**. A person having origins in any of the original peoples of the Far East, Southeast Asia, or the Indian subcontinent including, for example, Cambodia, China, India, Japan, Korea, Malaysia, Pakistan, the Philippine Islands, Thailand, and Vietnam.
- **Black or African American**. A person having origins in any of the black racial groups of Africa. Terms such as "Haitian" or "Negro" can be used in addition to "Black or African American."
- **Native Hawaiian or Other Pacific Islander**. A person having origins in any of the original peoples of Hawaii, Guam, Samoa, or other Pacific Islands.
- **White**. A person having origins in any of the original peoples of Europe, the Middle East, or North Africa.
- **Mixed Race**

*display if “Mixed race” is selected*

Please specify:

- __________

What is your ethnicity?

- Non-Hispanic
- Hispanic or Latino/a: A person of Cuban, Mexican, Puerto Rican, South or Central American, or other Spanish culture or origin, regardless of race. The term, "Spanish origin," can be used in addition to "Hispanic or Latino."

Is your household income from all sources:

- Less than $10,000
- $10,000 to less than $20,000
- $20,000 to less than $25,000
- $25,000 to less than $35,000
- $35,000 to less than $50,000
- $50,000 to less than $75,000
- More than $75,000
- Don’t know/Not sure
- Prefer not to answer

What is the highest grade or school you have completed?

- Never attended school or only attended kindergarten
- Grades 1 through 8 (Elementary)
- Grades 9 through 12, OR GED (High school graduate)
- College 1 year to 3 years (Some college or technical school)
- College 4 years or more (College graduate)
- Post-graduate education (Professional degree, Masters, Doctorate)
- Prefer not to answer

Before the COVID-19 pandemic, were you diagnosed with any of the following conditions? (check all that apply)

- None
- Thyroid disorder
- Asthma
- Chronic Obstructive Pulmonary Disease (COPD)
- Coccidioidomycosis (Valley Fever)
- Emphysema/Chronic bronchitis
- Seasonal allergies
- Diabetes
- Pre-diabetes
- Gestational diabetes
- Cancer (any type)
- Myocardial Infarction (heart attack)
- Heart disease such as angina (chest pain from heart problem)
- Congestive Heart Failure
- Stroke
- High blood pressure/hypertension
- High Cholesterol
- Other cardiac/heart disease
- Chronic liver disease
- Chronic kidney disease
- Inflammatory Bowel Disease (IBD) – Crohn’s Disease
- Inflammatory Bowel Disease (IBD) – Ulcerative Colitis
- Irritable Bowel Syndrome (IBS)
- Chronic diarrhea
- Chronic constipation
- Colitis (inflammation of the gut)
- Dyspepsia
- Reflux
- Clinically diagnosed depression or anxiety
- Parkinson’s
- Systemic Lupus (SLE)
- Multiple Sclerosis
- Rheumatoid Arthritis
- Other Arthritis
- Osteoporosis
- Other:___________________

What is your height?

Feet: __________ (ft)

Inches: __________ (in)

What is your weight?

- __________ (lbs)

In the month before becoming ill if you were sick, or in the previous month if you were not, were you a regular, occasional, or never smoker or vaper?

Cigarettes

- - Never
  - Occasionally
  - Regularly

Vaping (e-cigarettes) nicotine

- - Never
  - Occasionally
  - Regularly

Vaping Marijuana (wax pens or THC vapes)

- - Never
  - Occasionally
  - Regularly

Medical Marijuana

- - Never
  - Occasionally
  - Regularly

# Acute COVID-19 disease

On your last survey, you indicated you had not had any symptoms related to COVID-19 or tested positive for the virus. Have you experienced an illness that led you to believe you had COVID-19?

The most common symptoms of COVID-19 are fever, dry cough, and tiredness. Other symptoms that are less common and may affect some patients include aches and pains, nasal congestion, headache,

conjunctivitis, sore throat, diarrhea, loss of taste or smell or a rash on skin or discoloration of fingers or toes. These symptoms are usually mild and begin gradually. Some people become infected but only have very mild symptoms.

- Yes. I had symptoms.
- No. I have not had an illness like that
- Unsure

Have you tested positive for the virus that causes COVID-19 with a nasal swab, throat swab, or saliva?

- Yes – I was tested and got a POSTIVE test result
- Yes – I was tested and got a NEGATIVE test result
- Yes – I was tested but my results are pending
- No – I was not tested

*Display if “Yes, I tested positive” is selected*

What type of test was used to test you for COVID-19?

- Nasal swab (PCR test)
- Spit test (PCR test)
- Swab or spit RAPID test (results within a couple hours)
- Don’t know

*Display if “Yes – I was tested and got a POSTIVE test result” is selected*

What date were you tested for COVID-19? If you don't know the exact date, please choose your best approximation.

- __________ (mm/dd/yyyy)

Which of the following symptoms did you experience or are you currently experiencing? (Check all that apply)

- Elevated body temperature or Fever
- Cough
- Body aches/muscle pains
- Joint pain
- Changes in menstrual cycle
- Changes to vision
- Loss of smell/taste
- Confusion or brain fog (trouble concentrating)
- Dizziness or lightheadedness
- Headaches
- Sore Throat
- Congestion or runny nose
- Ringing in ears (tinnitus)
- Pink eye (conjunctivitis), red eyes, excessive tearing, or other eye symptoms

Lack of appetite

- Gastrointestinal symptoms (diarrhea, constipation, abdominal pain, nausea, vomiting, etc)
- Tightness in chest or chest pain
- Faster than normal heartrate
- Slower than normal heartrate
- High blood pressure (not previously diagnosed)
- Shortness of breath
- Fatigue (worn-out, low energy, difficulty or inability to complete daily activities)
- Insomnia or trouble sleeping
- Rash or other vascular issues (COVID toes, hive-like rashes, skin discoloration, bruising, etc)
- Post intensive care syndrome (PICS) (new or worsening impairment in physical health, thinking and judgment, or mental health status arising after an ICU stay and persisting after discharge)
- Stress and/or anxiety

On a scale from 1-10, how would you characterize the severity of your illness?

1 - very mild easy to continue daily activities to 10 - extremely serious

- 0
- 1
- 2
- 3
- 4
- 5
- 6
- 7
- 8
- 9
- 10

Were you hospitalized for this illness?

- Yes
- No
- Unknown

# Post-acute COVID-19 disease

Have you noticed any of the following ongoing or new symptoms?

- None
- Chills or sweats
- Elevated body temperature or Fever
- Cough
- Body aches/muscle pains
- Joint pain
- Changes in menstrual cycle
- Changes to vision
- Loss of smell/taste
- Confusion or brain fog (trouble concentrating)
- Dizziness or lightheadedness
- Headaches
- Sore Throat
- Congestion or runny nose
- Ringing in ears (tinnitus)
- Pink eye (conjunctivitis), red eyes, excessive tearing, or other eye symptoms
- Fatigue
- Lack of appetite
- gastrointestinal symptoms (diarrhea, constipation, abdominal pain, nausea, vomiting, etc)
- Tightness in chest or chest pain
- Faster than normal heartrate
- Slower than normal heartrate
- High blood pressure (not previously diagnosed)
- Shortness of breath
- Fatigue (worn-out, low energy, difficulty or inability to complete daily activities)
- Insomnia or trouble sleeping
- Rash or other vascular issues (COVID toes, hive-like rashes, skin discoloration, bruising, etc)
- Post intensive care syndrome (PICS) (new or worsening impairment in physical health, thinking and judgment, or mental health status arising after an ICU stay and persisting after discharge)
- Stress and/or anxiety
- Other

We know that the above was not an exhaustive list of all the symptoms that people experience over the course of their illness. Would you share with us any other new symptoms you started noticing that were not included in the above list?

- __________

Have any of your symptoms gone away for more than one day and then come back?

- Yes
- No

Please select the symptoms that went away and then came back during your illness.

- None
- Chills or sweats
- Elevated body temperature or Fever
- Cough
- Body aches/muscle pains
- Joint pain
- Changes in menstrual cycle
- Changes to vision
- Loss of smell/taste
- Confusion or brain fog (trouble concentrating)
- Dizziness or lightheadedness
- Headaches
- Sore Throat
- Congestion or runny nose
- Ringing in ears (tinnitus)
- Pink eye (conjunctivitis), red eyes, excessive tearing, or other eye symptoms
- Fatigue
- Lack of appetite
- gastrointestinal symptoms (diarrhea, constipation, abdominal pain, nausea, vomiting, etc)
- Tightness in chest or chest pain
- Faster than normal heartrate
- Slower than normal heartrate
- High blood pressure (not previously diagnosed)
- Shortness of breath
- Fatigue (worn-out, low energy, difficulty or inability to complete daily activities)
- Insomnia or trouble sleeping
- Rash or other vascular issues (COVID toes, hive-like rashes, skin discoloration, bruising, etc)
- Post intensive care syndrome (PICS) (new or worsening impairment in physical health, thinking and judgment, or mental health status arising after an ICU stay and persisting after discharge)
- Stress and/or anxiety
- Other

*Display if “other” is selected.*

Please explain the other symptoms you are referring to.

- __________
